# A plethora of techniques to detect mutations: Which one to choose?

**DOI:** 10.4103/0971-6866.44102

**Published:** 2008

**Authors:** K. Ghosh, S. Shetty

**Affiliations:** National Institute of Immunohaematology, 13^th^ Floor, New Multistoried Bldg, K.E.M. Hospital Campus, Parel, Mumbai - 400 012, India

In late eighties and early nineties detection of mutations in a gene involved painstaking long polyacrylamide gel electrophoresis followed by autoradiography utilizing dideoxy nucleotide termination technique of Sanger.[1] This was subsequently followed by several mutation screening techniques like DGGE, TGGE, SSCP, CSGE and various other modifications of heteroduplex analysis. Soon enough the deficits and advantages of these techniques were also described.[2] Simultaneously, various automated sequencing techniques were also evolved (i.e., dideoxy, pyrophosphate, dHPLC and so on) as seen by the virtual hurricane of published reports on mutations in different genes.

The latest development in these techniques is the evolution of microarray technique which facilitates the screening of large number of genes on a small piece of glass slide within a short span of time. This has facilitated the detection of single nucleotide polymorphisms (SNPs) as well as the smaller deletions and point mutations.

In the present issue, Massimilliano used a modification of microarray technique utilizing fluorescent labeled random hexamers on a solid phase along with fluorescent labeled pentamers as the hybridizing oligonucleotides. Finally, when the random hexamers and pentamers in the given dots coincided with the complementary sequences of the amplified PCR product, they get ligated by the DNA ligase present in the reaction mixture. Using appropriate software and the principle of combinational analysis, the sequence of the fragments were determined.

This is an open platform of random hexamers and pentamers and is likely to have universal application. The investigators could detect known deletions, nonsense and missense mutations of factor VIII gene in all the cases. Moreover, it also detected that mutations in a few cases where previous screening for mutations was negative showing the robustness and sensitivity of the test.

However, the test is unlikely to be cheap for its utility in developing countries like India, where one of the major objectives of mutation detection is for carrier diagnosis and prenatal diagnosis. In case of haemophilia A, we have used a combination of different techniques like intron 22 and 1 inversion and RFLP analysis.[3] We also had to take recourse to phenotypic diagnosis due to the noninformativeness of the markers or due to the absence of key members in the family.[4]

The technique presented here for the detection of factor VIII gene mutations requires only three days to complete the scanning of entire factor VIII gene. However, one should not loose sight of the fact that they have failed to get mutations in quite a few cases. This is an addition to already existing list of screening techniques. It is up to the investigator to choose the most appropriate ones from this battery of techniques to give an accurate, cost effective diagnosis to the affected families.

From our own experience and experience of others in the area of GPIIb IIIa, mutations for Glanzmann's Thrombosthenia (GT), we have found that even after using a mixture of technologies like CSGE,SSCP, DGGE and whole exon sequencing, we are still not able to find mutations in 30-40% of the cases. Going by the report on the technique under discussion it seems that the technique will allow us to find more mutations in GT patients or for other genetic disorders where complex and big genes are involved.
